# An *in vitro* single-molecule assay for eukaryotic cap-dependent translation initiation kinetics

**DOI:** 10.1093/nar/gkz1066

**Published:** 2019-11-13

**Authors:** Hongyun Wang, Lexi Sun, Anthony Gaba, Xiaohui Qu

**Affiliations:** 1 Molecular Biology Program, Memorial Sloan Kettering Cancer Center, New York, NY 10065, USA; 2 Department of Physiology, Biophysics and Systems Biology, Weill Cornell Graduate School of Medical Sciences, Cornell University, New York, NY 10065, USA

## Abstract

Eukaryotic mRNAs are predominantly translated via the cap-dependent pathway. Initiation is a rate-limiting step in cap-dependent translation and is the main target of translational control mechanisms. There is a lack of high-resolution techniques for characterizing the cap-dependent initiation kinetics. Here, we report an *in vitro* single-molecule assay that allows characterization of both initiation and peptide chain elongation kinetics for cap-dependent translation. Surprisingly, the histogram of the first-round initiation time is highly asymmetrical and spans a large time range that is several-fold greater than the average peptide synthesis time in translation reactions with a firefly luciferase-encoding mRNA. Both the histogram and single-molecule trajectories reveal an unexpected high-degree of asynchrony in translation activity between mRNA molecules. Furthermore, by inserting a small stem-loop (Δ*G* = −4.8 kcal/mol) in the middle of the mRNA 5′ untranslated region (UTR), our assay robustly detects small changes in budding yeast initiation kinetics, which could not be resolved by bulk luminescence kinetics. Lastly, we demonstrate the general applicability of this assay to distinct cell-free translation systems by using extracts prepared from budding yeast, wheat germ, and rabbit reticulocyte lysates. This assay should facilitate mechanistic studies of eukaryotic cap-dependent translation initiation and translational control.

## INTRODUCTION

Cap-dependent translation is the predominant pathway for eukaryotic translation (1–4). Initiation is a rate-limiting step in cap-dependent translation (1,2) and the main target of translational control mechanisms (2–4). Genetic (5), biochemical (6–8), structural (9) and genomic-scale approaches (10) have greatly advanced our understanding of cap-dependent initiation mechanisms. However, kinetic characterization is still limited. Various approaches were developed for measuring the overall progression of the translation process, including luciferase- (11,12) and SNAP-based (13) assays. These approaches all detect the synthesis of large protein products. Recently, several *in vivo* fluorescence assays were developed to measure cellular translation kinetics based on fluorescent antibody binding to epitopes in nascent peptides (14–17). Due to the high fluorescent background in cells, an mRNA engaged in active translation was detected when bound with multiple antibodies. All these existing approaches lack high resolution for measuring the kinetics of individual initiation events, although the average initiation rate can often be estimated from the experimental observables by mathematical modeling.

Being able to track individual initiation events will provide a high-resolution kinetic lens for studying cap-dependent initiation mechanisms, especially when used in combination with mutations in the translation machinery or mRNA. *In vitro* single-molecule techniques are good candidates for developing such assays. However, despite their great success with prokaryotic translation (18–22), the application of *in vitro* single-molecule techniques to eukaryotic translation has been limited to the studies of individual initiation factor interactions in the absence of active translation (23–27), IRES-mediated initiation (28,29), and peptide chain elongation (30–32). An *in vitro* single-molecule condition suitable for studying the cap-dependent initiation pathway has not been reported.

**Figure 1.**
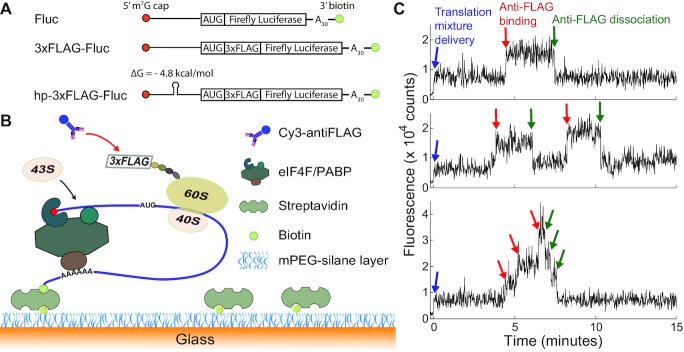
Overview of the *in vitro* single-molecule assay. (**A**) The sequence design of Fluc, 3xFLAG-Fluc, and hp-3xFLAG-Fluc mRNAs. Each mRNA is 5′ capped, 3′ polyadenylated, and 3′ biotinylated. (**B**) Schematic of the single-molecule assay. Translation mixture supplemented with Cy3 labeled anti-FLAG is introduced into the flow channel to translate the 3′ end anchored reporter mRNAs. When the N-terminal 3xFLAG tag on the nascent peptide emerges from the exit tunnel of a translating ribosome, Cy3-antiFLAG binds to the 3xFLAG tag and the binding is detected by TIRF imaging. (**C**) Three representative single molecule trajectories for yeast extract (YE) translation of individual 3xFLAG-Fluc mRNAs. The early increase in baseline counts, denoted by the blue arrows, results from the delivery of YE/Cy3-antiFLAG into the detection channel. This increase sets the starting point (time 0) of the translation reaction. Individual Cy3-antiFLAG binding to a nascent peptide results in an instantaneous increase in fluorescence signal, as indicated by the red arrows. The dissociation of individual antibody/nascent peptide complexes upon translation termination leads to an instantaneous decrease in fluorescence signal, indicated by the green arrows.

Here, we report the first *in vitro* single-molecule assay that allows kinetic characterization of individual cap-dependent initiation events. Our assay is based on single-molecule fluorescence imaging of Cy3-labeled anti-FLAG binding to nascent N-terminal 3xFLAG tag peptides during active translation (Figure 1). Antibody binding to the N-terminal tag, which occurs shortly after initiation, allowed us to track initiation kinetics with single-molecule resolution. This assay also robustly detected distinct initiation kinetics resulting from the insertion of a small stem-loop structure in the middle of a reporter mRNA’s 5′ untranslated region (UTR). The modest effects of the small stem-loop could not be resolved by bulk kinetic measurements. Furthermore, we successfully implemented this assay with three cell lysate-based translation systems, demonstrating the general applicability of this assay to fungal, plant, and mammalian *in vitro* translation systems.

## MATERIALS AND METHODS

### RNA synthesis

The mRNA sequences are described in the Results section on ‘Overview of the single-molecule assay’. The plasmid encoding the Fluc mRNA under the control of the T7 promoter, plasmid linearization, *in vitro* transcription, and RNA purification were as described previously (33). To insert the 3xFLAG sequence after the firefly luciferase ATG start codon, the Fluc plasmid was digested by NcoI (NEB) and KasI (NEB), which cut 1 nt before the ATG and in the middle of the coding region, respectively. The cut sequence, with an insertion of the 3xFLAG sequence (GACTAC AAAGAC CATGAC GGTGAT TATAAA GATCAT GATATC GATTAC AAGGAT GACGAT GACAAG) after ATG, was ligated back to the Fluc plasmid backbone. To insert the short stem-loop in the middle of the 5′ UTR, the 3xFLAG-Fluc plasmid was cut with BglII (NEB) and NcoI (NEB) to delete the entire 5′UTR. Then the 5′UTR with an insertion of a hairpin-forming sequence (GCCGATATCACGGC) at the 90 nt position from the mRNA 5′ end was ligated back into the 3xFLAG-Fluc plasmid backbone. Synthesis of the 3xFLAG-Fluc and hp-3xFLAG-Fluc mRNAs followed the same procedure as for the Fluc mRNA. Plasmids and their sequences are available upon request. The synthesized mRNAs were capped using a Vaccinia Capping System (NEB) and 3′ biotinylated using the Pierce RNA 3′ End Biotinylation Kit, performed according to the manufacturer's recommended protocol. After capping and biotinylation, the mRNAs were purified with phenol-chloroform extraction and the Direct-Zol RNA kit (Zymo Research), from which the RNAs were eluted in H_2_O. The quality and concentration of all mRNAs were checked using 5% acrylamide 8M urea 1x TBE gels, as described previously (33). All the mRNAs were stored at −80°C.

### YE preparation, bulk luciferase activity assay, and translation reagents for single-molecule detection

The budding yeast translation extract (YE) was prepared from *S. cerevisiae* strain YAS1874 (*MAT***a***MAK10::URA3 PEP4::HIS3 prb1 prc1 ade2 trp1 his3 ura3*) (34) as described by Wu *et al.* (35) with the following modifications: (i) yeast cultures were grown until OD_600_ ≈ 3.5, (ii) buffer A was at pH 7.4, (iii) lysates were clarified by two 15 min spins at 16 000 rpm in a SS-34 rotor and (iv) small molecules were removed from the supernatant with Zeba Desalt Spin Columns (Pierce), following the manufacturer's protocol and using buffer A for pre-equilibration.

Bulk translation reactions were performed as described by Wu *et al.* (35) with minor modifications. A typical translation reaction contained 50% by volume of yeast extract, 35.06 mM HEPES/KOH (pH 7.4), 160 mM KOAc, 3.5 mM Mg(OAc)_2_, 1 mM ATP, 0.1 mM GTP, 20 mM phosphocreatine, 0.06 U/μl creatine phosphokinase, 10 uM of each of the 20 amino acids, 3.166 mM DTT, 0.25 mM PMSF, 0.8 U/μl RNase inhibitor, and the RNA substrate (typically 0.4 ng/μl). Translation reactions were at 25°C for 60 min and terminated by freezing in liquid N_2._ To measure luciferase activity, ice-thawed reaction mixtures were diluted with an equal volume of 2× passive lysis buffer (Promega) and 5 μl aliquots of the mixture were used with 50 μl of luciferase assay reagent (Promega) for luminescence measurements on a GloMax 96 Microplate Luminometer. For kinetic luminescence measurements, translation reactions were assembled as described above and additionally supplemented with 0.5 mM Luciferin (Prolum Ltd) and 0.1 mM Co-enzyme A (Prolume Ltd) (36,37). Typically, 20 μl of cell-free translation reaction was loaded per well on the GloMax 96 Microplate Luminometer, and bioluminescence was continuously measured for up to 15 min with a time resolution of 1 or 10 s.

For the single–molecule experiments, the translation mixture was assembled as described above but without mRNA. This mixture was supplemented with 67 nM of Cy3-antiFLAG (Sigma A9594), except when specified otherwise. For the measurements of anti-FLAG binding to pre-existing nascent 3xFLAG peptides, 4 mM cycloheximide (Sigma C7698) was included in the YE/Cy3-antiFLAG translation mixture.

### WGE and RRL translation mixtures for single-molecule detection

Wheat germ extract (WGE) was from Promega (L4380). Translation reaction mixtures for single-molecule detection were assembled according to the manufacturer's instructions with the following modifications: (i) the RNase inhibitor RNasin (Promega) and amino acids were used at a final concentration of 0.4 U/ul and 10 uM per amino acid, respectively; (ii) the KOAc concentration was adjusted to 103 mM for optimal translation activity; (iii) no mRNA was added and (iv) Cy3-antiFLAG was added to a final concentration of 134 nM.

Rabbit Reticulocyte Lysate (RRL) was from Promega (L4960). Translation reaction mixtures for single-molecule detection were assembled according to the manufacturer's instructions except that (i) no mRNA was added and (ii) Cy3-antiFLAG was added to a final concentration of 67 nM.

### Construction of the single-molecule detection chamber

A No. 1.5 coverslip and a glass slide were cleaned, silanized, and PEGylated as described by Jain *et al.* (38) with modifications: (i) the cleaning was done sequentially in 10% (v/v) alkaline liquid detergent for 20 min with sonication, Piranha solution for 60 min, 1 M KOH for 20 min with sonication, and methanol for 20 min with sonication; (ii) the silanization reaction was for 20 min in total with a 1-min sonication after the initial 10 min. For the double PEGylated surface, a second round of PEGylation with MS(PEG)_4_ (Thermo Fisher scientific) was performed and immediately followed by assembly of the single-molecule chamber, as described by Chandradoss *et al.* (39). The flow channels were constructed between the coverslip and microscope slide with double-sided tape, as described by Jain *et al.* (38). The volume of each flow channel is ∼7 ul.

### Single molecule imaging and data analysis

The flow channel was first incubated with 0.2 mg/ml streptavidin (Thermo scientific) for 10 min and then washed three times, each with 20 ul of T50 buffer (20 mM Tris–HCl, pH 7.0, 50 mM NaCl). The 3′end biotinylated reporter mRNAs in T50 buffer were added to the flow chamber, incubated for 15 min, and washed with translation buffer (35.06 mM HEPES/KOH pH 7.6, 160 mM KOAc, 3.5 mM Mg(OAc)_2_ for YE; 53 mM KOAc and 2.1 mM Mg(OAc)_2_ for WGE; 79 mM KOAc and 0.5 mM Mg(OAc)_2_ for RRL) to remove all unbound mRNAs. The concentration of the reporter mRNA (typically 1–4 ng/ul) was adjusted to allow single-molecule density of antibody binding. A few seconds after data acquisition starts, 20 ul of the selected translation mixture was delivered into the channel through a home-built microfluidic adaptor by a Harvard Apparatus syringe pump at a speed of 150 ul/min. At the end of single-molecule detection, typically for 1 h, the translation mixture was pipetted out of the flow channel and supplemented with luciferase activity assay reagents to measure the luminescence. All the single-molecule experiments were carried out at room temperature of 22–23°C.

Objective-type TIRF imaging was carried out on an Olympus IX83 inverted microscope equipped with a 100× oil immersion objective (N.A. 1.49), 100 mW 532 nm laser with adjustable output, CellTIRF illuminator, Andor iXon Ultra 897 EMCCD, Chroma 532/640/25 excitation filter, Semrock R405/488/532/635 dichroic, and Semrock NF03-405/488/532/635E-25 emission filter. Absorptive-type neutral density filters were installed in the CellTIRF unit to further reduce the laser illumination intensity when necessary. Data were recorded as a kinetic series at the speed of 0.5 to 2 s per frame.

Under the commonly used TIRF imaging condition, the laser incident angle is just slightly above the critical angle (63.8° for our experimental setup). Under this condition, the high concentration of diffusing fluorescent antibodies in our experiments gives rise to a very high fluorescent background, which washes out the fluorescence signal from single antibody binding to surface immobilized mRNA/ribosome/peptide complexes. To significantly reduce the fluorescent background to allow single-molecule detection, we used a larger laser incident angle of 71.5°.

Image analysis was performed using custom written Matlab codes. Each movie was drift and background corrected. The bound antibodies in each frame were identified based on their fluorescence intensities. Antibody positions were determined with sub-pixel resolution by 2D Gaussian fitting of their intensity profiles (40).

## RESULTS

### Overview of the single-molecule assay

The designs of mRNA constructs used in our studies are shown in Figure 1A. We used a Firefly luciferase encoding mRNA (‘Fluc’ mRNA) as a model mRNA to facilitate the comparison between single-molecule and bulk luminescence measurements. The Fluc mRNA is composed of a 177 nucleotide (nt) 5′ UTR, 1656 nt coding region, a 12 nt 3′ UTR, and a 30 nt poly(A) tail. This mRNA was used previously for studying arginine-dependent translational control mechanisms (41,42). We chose to base our assay on anti-FLAG and 3xFLAG peptide interaction due to its superior sensitivity and specificity in comparison to other commonly used antibody/epitope interactions (43). Accordingly, a 3xFLAG-encoding sequence was inserted into the Fluc mRNA immediately after the AUG start codon to generate ‘3xFLAG-Fluc’ mRNA. To moderately perturb the initiation kinetics, we inserted a small stem–loop (hairpin) structure (sequence GCCGATATCACGGC; the stem forming bases are underlined) into the 3xFLAG-Fluc mRNA 5′ UTR, positioned 90 nt from the 5′ mRNA terminus to generate ‘hp-3xFLAG-Fluc’ mRNA. All mRNAs were capped at the 5′ terminus to allow cap-dependent translation and biotinylated at the 3′ terminus to enable mRNA surface immobilization for single-molecule imaging.

In the assay (Figure 1B), mRNAs are tethered via their 3′ ends to a streptavidin-coated single-molecule detection surface and unbound mRNAs are washed away. Data acquisition starts with only mRNAs on the surface. Shortly afterwards, an *in vitro* translation system supplemented with Cy3-labeled anti-FLAG is introduced into the flow channel to allow translation. After the translation of ∼30–40 codons (44,45) downstream of the 3xFLAG sequence, the 3xFLAG tag on the nascent peptide will emerge from the ribosome exit tunnel and be accessible to anti-FLAG binding. The antibody binding to the nascent peptide is detected by a Total Internal Reflection Fluorescence (TIRF) microscope in real-time and at single-molecule resolution. After single-molecule detection, the translation mixture is removed from the flow channel and is used for measurement of luciferase enzymatic activity to determine the level of synthesized luciferase proteins.

Comparison of luminescence kinetics in yeast extract (YE) translation reactions with 3xFLAG-Fluc mRNA in microcentrifuge tubes (gray and green) vs. in the single-molecule chambers (red) indicated that the single-molecule conditions preserved the translation kinetics (Supplementary Figure S1). Example single-molecule trajectories for YE translation of this mRNA are shown in Figure 1C. Each trajectory represents the detected fluorescence change for a single mRNA molecule. The delivery of translation reagent at the beginning gives rise to a baseline increase due to the diffusing fluorescent antibodies (blue arrows). The reagent exchange occurs simultaneously for all trajectories from the same field of view. For our flow chamber configuration and typical flow delivery speed (Materials and Methods), the reagent exchange takes ∼3–4 s to complete (Supplementary Figure S2). The completion of reagent exchange sets the starting point of the translation reaction and is therefore marked as time 0 in the analysis of translation kinetics. Each antibody binding to the translating ribosome/mRNA/peptide complex gives rise to an instantaneous fluorescence increase (red arrows), while antibody dissociation leads to an instantaneous fluorescence decrease (green arrows). The timing of anti-FLAG binding and dissociation differs between trajectories as all molecular interactions are intrinsically stochastic on the single-molecule level. Measurement of the time lag between the delivery of translation reagent and the first antibody binding enables tracking of the first-round of initiation on single mRNA molecules.

### Specificity of anti-FLAG binding to nascent 3xFLAG tags

A low level of nonspecific antibody binding is desirable in this assay. The experiment for characterizing nonspecific binding is similar to the translation initiation assay illustrated in Figure 1B, except that a 3xFLAG-lacking Fluc mRNA is used. We measured the level of nonspecific antibody binding under various 532 nm laser illumination intensities and also compared flow channels that were passivated using either the single-round (38) or double-round (39) surface PEGylation protocol (Materials and Methods). With both surface PEGylation protocols, the nonspecific binding level increased with the 532 nm laser illumination (Supplementary Figure S3). With up to 10 μW excitation measured at the objective, the two surfaces performed similarly. However, under higher laser excitation, the single-round PEGylated surface (Supplementary Figure S3 blue) showed 2–3-fold higher nonspecific binding than the double-round PEGylated surface (Supplementary Figure S3 red). Based on these observations, all subsequent YE experiments were performed with the double PEGylated surface and 10 μW laser power to maintain low levels of nonspecific antibody binding.

**Figure 2.**
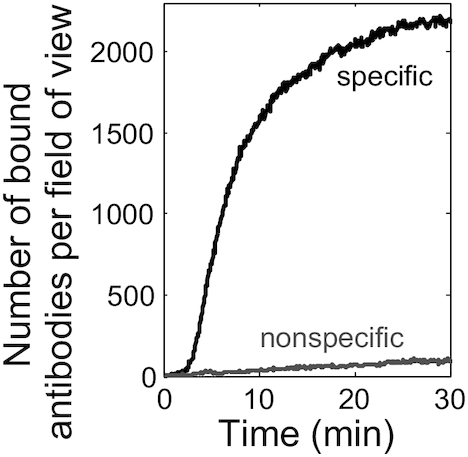
Specificity of Cy3-antiFLAG binding to nascent 3xFLAG peptides. Representative time courses of the number of Cy3-antiFLAG binding events in YE translation of Fluc (gray) and 3xFLAG-Fluc (black) mRNAs. Data are representative of 16 experiments for specific binding and 5 experiments for nonspecific binding.

To compare the levels of nonspecific vs. specific binding, immobilized Fluc and 3xFLAG-Fluc mRNAs were translated in adjacent flow channels. To increase the sensitivity of detecting nonspecific antibody binding, immobilized Fluc mRNA was 2.3-fold more concentrated than immobilized 3xFLAG-Fluc mRNA. The luminescence readings after single-molecule detection were proportionally 2-fold greater for Fluc than 3xFLAG-Fluc mRNA, indicating that the two mRNAs were translated with comparable efficiency. However, the time-resolved kinetics of the number of antibody binding events per field of view were very different between the two channels over a 30-minute translation reaction (Figure 2). The number of nonspecifically bound antibodies (gray) remained low and showed an approximately linear increase over time, indicating a continuous accumulation during data acquisition. In contrast, specific binding (black) started to appear around 2 minutes and then continually increased toward a high-level plateau. This observation agrees well with the expected kinetics of nascent peptide synthesis, which should have an early lag due to the time required for initiation and a subsequent steady-state with dynamic equilibrium between newly initiated peptide synthesis and peptide release upon translation termination. The large differences in anti-FLAG binding levels and kinetics with the Fluc and 3xFLAG-Fluc mRNAs demonstrated that the Cy3-antiFLAG binds specifically to the nascent N-terminal 3xFLAG tag under our single-molecule conditions. For YE translation, there was typically at least a 20-fold difference for the specific versus nonspecific binding ratio (‘*SNBR*’), calculated when the level of specific binding reaches the plateau (Figure 2).

### Kinetics of anti-FLAG recognition of nascent 3xFLAG peptides

**Figure 3.**
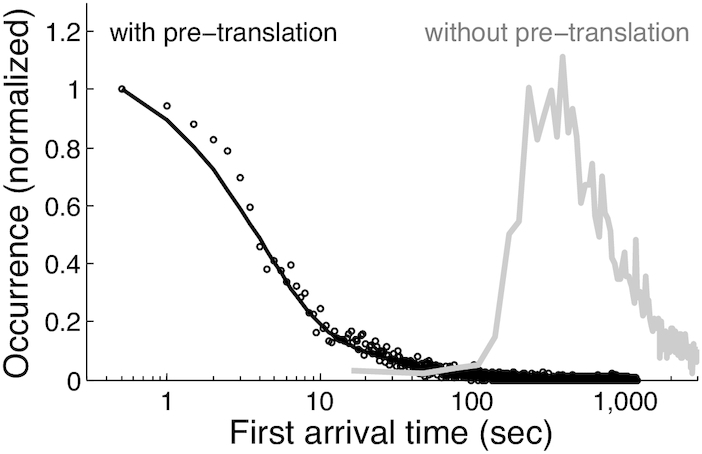
Histograms of first arrival time for Cy3-antiFLAG binding with (black) and without (gray) pre-translation. Black: YE translation of 3′-end tethered 3xFLAG-Fluc mRNA was used to generate ribosome/nascent peptide/mRNA complexes on the detection surface. YE/Cy3-antiFLAG was then flowed into the channel and antiFLAG binding to pre-existing nascent 3xFLAG peptides was measured. *n* = 4516 trajectories. The first arrival time histogram (black circles) fits reasonably well to a double exponential distribution (black curve): $y = {A_1}/{\tau _1} \cdot {e^{ - t/{\tau _1}}} + {A_2}/{\tau _2} \cdot {e^{ - t/{\tau _2}}}$, with *τ*_1_ = 3.9 ± 0.2 (s.e.) s, *τ*_2_ = 38 ± 2 (s.e.) s, and *A*_1_*/A*_2_= 13 ± 1(s.e.). A boot-strapping method was used for fittings, as described previously (54). Gray: YE/Cy3-antiFLAG was delivered into the flow channel immediately after mRNA surface immobilization and antibody binding was measured during real-time translation. *n* = 2728 trajectories. The x-axis is in log scale for better visualization because antibody binding under these two conditions occur on very different time scales. The same data and fit result for the condition with pre-translation are shown again in linear scale in Supplementary Figure S4A.

In order to achieve a high resolution for initiation kinetics, antibody recognition of nascent 3xFLAG peptides needs to be much faster than initiation. To measure antibody binding time, we first used YE to translate 3′ end-tethered 3xFLAG-Fluc mRNAs and generated ribosome/mRNA/nascent peptide complexes on the single-molecule surface. After 20 min of translation, we flowed in YE/Cy3-antiFLAG and measured anti-FLAG binding to pre-existing 3xFLAG nascent peptides. To determine the binding kinetics we analyzed the first arrival time (*t*_1_), which corresponds to the time lag from the completion of reagent exchange to the first antibody binding per mRNA. The histogram of *t*_1_ for 67 nM antibody with pre-translation (Figure 3 black circles) fits a double-exponential distribution with time constants *τ*_1_ = 3.9 ± 0.2 (s.e.) s and *τ*_2_ = 38 ± 2 (s.e.) s (Figures 3 black curve and S4A). The ratio of the amplitude of the fast vs. slow exponential component is 13 ± 1 (s.e.), indicating that 92.8% ± 0.5% (s.e.) of antibody binding events occurred with the fast rate constant. To suppress further peptide elongation, the YE/Cy3-antiFLAG was flowed in together with 4 mM of the peptide elongation inhibitor cycloheximide (CHX), which is insufficiently effective to stall all translating ribosomes in 4 s (46). Therefore, we made the following assignments: (i) a fast exponential component for antibody binding to pre-existing 3xFLAG peptides that were completely out of the ribosome exit tunnel and (ii) a slow exponential component for nascent peptide chains that needed to be further extended to make the 3xFLAG tag accessible to antibody binding. Similar analyses for lower antibody concentrations of 22 and 7 nM resulted in time constants of 5.6 ± 0.5 (s.e.) s and 64 ± 7 (s.e.) s, respectively, for anti-FLAG recognition of accessible 3xFLAG nascent peptides (Supplementary Figure S4B and C).

Figure 3 (gray curve) shows the histogram of *t*_1_ for YE translation in the presence of 67 nM antibody but without pre-translation. Consistent with Figure 2, the histogram of *t*_1_ started to populate at ∼2 min and spanned a large time range up to ∼20 min. Therefore, even the fastest peptide synthesis events started after 2 min of translation reaction and were at least 20× slower than the <6 s time constants for 3xFLAG epitope recognition for the 22 nM or higher antibody concentrations. Collectively, these results indicate that antibody binding is not rate-limiting for detecting initiation kinetics under these conditions. All subsequent YE translation experiments were performed with 67 nM Cy3-antiFLAG. TIRF imaging conditions were adjusted to accommodate the high antibody concentration and allow single-molecule detection, as described in Materials and Methods.

### Initiation kinetics via first arrival time analysis of antibody binding

**Figure 4.**
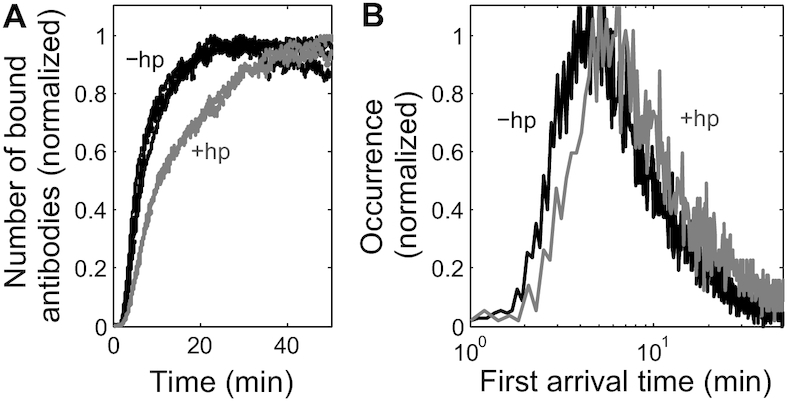
A small hairpin structure in the 5′ UTR of 3xFLAG-Fluc mRNA slows initiation. (**A**) Time courses of the number of antibody binding in YE translation of 3xFLAG-Fluc (black; 3 data sets) and hp-3xFLAG-Fluc (gray; 2 data sets) mRNAs, respectively. The three black data sets and two gray data sets overlay significantly within each group, indicating the robustness of this measurement. (**B**) The corresponding first arrival time histograms of the experiments in (A). The multiple data sets for each mRNA from (A) were pooled together in (B). *n* = 10 650 trajectories for -hp mRNA and 4079 trajectories for +hp mRNA. The x-axis is in log scale for better visualization of the difference in first arrival time between the two mRNAs. Both histograms fit well to the shifted (3-parameter) log-normal distribution (Equation 1, Supplementary Figure S6, and Supplementary Table S1).

Since Cy3-antiFLAG binds to nascent 3xFLAG peptides in a fast and specific manner under our experimental conditions, antibody binding should be a good tracker of the initiation progress. To test the sensitivity of this assay for measuring initiation kinetics, we compared the translation of 3xFLAG-Fluc (‘-hp') versus hp-3xFLAG-Fluc (‘+hp') mRNAs (Figure 1A). The inserted stem-loop contains 4 G·C base pairs in the stem and a 6 nt loop, yielding a thermostability of –4.8 kcal/mol as calculated by Mfold (47). Previous bulk studies suggested that such hairpins were too weak to affect YE translation (48). Indeed, we were unable to resolve differences in translation kinetics with the –hp and +hp mRNAs by bulk kinetic luciferase activity assay (Supplementary Figure S5; *n* = 9 and 6 repeats for -hp and +hp mRNA, respectively). In contrast, with our single-molecule assay we observed slower antibody binding with +hp mRNA than with –hp mRNA (Figure 4A; *n* = 3 and 2 repeats for –hp and +hp mRNA, respectively). The multiple repeats of each condition overlaid within each group, indicating that our assay was highly reproducible under these experimental conditions. The observed difference in antibody binding rate between the two mRNAs was well above the experimental variations between the multiple data sets for each mRNA. These data demonstrate that the inserted hairpin structure perturbed the initiation kinetics and indicate that the single-molecule assay has a higher sensitivity for measuring changes in initiation kinetics than the bulk luminescence kinetic assay.

Translation is a very dynamic process on the single-molecule level as demonstrated by representative single-molecule trajectories for YE translation (Figure 1C). Among the rich kinetic information that the trajectories contain for initiation, the first arrival time (*t*_1_) is biochemically defined as the sum of the first-round initiation time (*t*_1_I_) and the peptide chain elongation time for synthesizing the 3xFLAG tag and the subsequent 30–40 amino acids (*t*_1_tag_). In contrast, other kinetic parameters, such as the timing of the subsequent antibody binding events, will depend on some poorly understood molecular interactions, including the duration that the first recruited small ribosomal particle sequesters the mRNA from subsequent small ribosomal particle binding. We therefore focused on analyzing the antibody first arrival time to quantify initiation kinetics. The Cy3-antiFLAG first arrival time histograms for +hp and –hp mRNAs showed that the inserted stem-loop structure increased the antibody first arrival time (Figure 4B), consistent with slower antibody binding in translation reactions with the +hp mRNA (Figure 4A).

Among commonly used functional forms for curve fitting, the distribution of *t*_1_ for both mRNAs fit well to the shifted (three-parameter) log-normal distribution (49) (Supplementary Figure S6A and B, Supplementary Table S1):

(1) $$\begin{equation*}y = \left\{ {\begin{array}{@{}*{3}{c}@{}} {{y_0}}&,&{x \le {x_0}}\\ {{y_0} + \frac{A}{{\sqrt {2\pi } \cdot \sigma \cdot \left( {x - {x_0}} \right)}} \cdot {e^{ - {{\left( {ln\left( {x - {x_0}} \right) - \mu } \right)}^2}/\left( {2{\sigma ^2}} \right)}}}&,&{x > {x_0}} \end{array}} \right.\end{equation*}$$

The above function differs from the standard shifted log-normal function only in *y*_0_, which is added to account for nonspecific antibody binding. From the fitting results, the 5′ UTR hairpin slowed initiation by 1.3 ± 0.9 (s.e.) min, measured by the peak position of the histogram, or 2.7 ± 2.4 (s.e.) min, measured by the mean of the first arrival time.

### Peptide chain elongation kinetics via dwell time analysis of antibody binding

**Figure 5.**
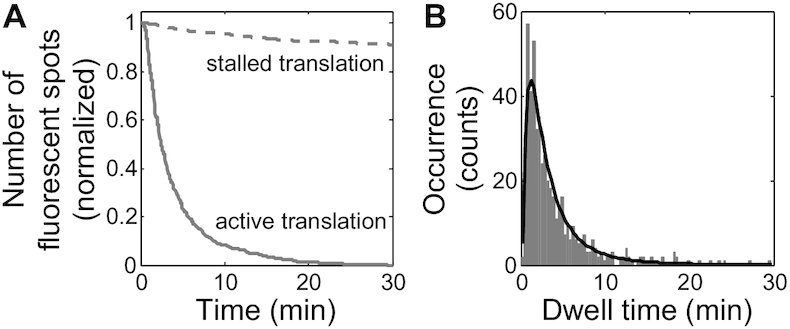
Dwell time analysis of antibody binding for YE translation. (**A**) Survival probability of fluorescent spots during stalled (dashed) and active (solid) translation. Cy3-antiFLAG/peptide/ribosome/mRNA complexes were generated on flow channel detection surfaces by YE/Cy3-antiFLAG translation of 3′ end tethered 3xFLAG-Fluc mRNAs. Loss of fluorescent spots over time was then measured in the presence of translation buffer (stalled translation) or YE (active translation). *n* = 1998 trajectories for stalled translation and 515 trajectories for active translation. (**B**) Histogram of the dwell times of Cy3-antiFLAG binding during active YE translation of 3xFLAG-Fluc mRNA. *n* = 515 trajectories. Log-normal distribution fitting (black curve) yielded an average peptide chain elongation time of 3.9 ± 0.2 (s.e.) min, which corresponds to a peptide chain elongation rate of 2.5 ± 0.1 (s.e.) amino acids per second.

The loss of fluorescence signal on single-molecule trajectories (Figure 1C) can be used to track peptide release upon translation termination and ribosomal subunit dissociation. This analysis is possible if the total decoding time is much shorter than the time scale of fluorescence loss due to translation-irrelevant events, such as Cy3-antiFLAG dissociation from the 3xFLAG epitope or fluorophore photobleaching. To measure fluorescence loss caused by such translation-irrelevant events, we generated stalled ribosome/mRNA/peptide/Cy3-antiFLAG complexes on the single-molecule surface by first incubating 3′ end-tethered 3xFLAG-Fluc mRNA with YE/Cy3-antiFLAG and then washing the channel with translation buffer. The translation buffer has the same ionic strength as YE does to stabilize the ribosome/mRNA/peptide complex, but lacks tRNAs and elongation factors required for more rounds of peptide chain elongation. After 30 min of stalled ribosome complex incubation in translation buffer with laser excitation, we observed an ∼10% loss of fluorescent spots (Figure 5A, dashed line). By contrast, after only 10 min of active YE translation, >90% of the fluorescent spots were lost (Figure 5A, solid line). Therefore, our experimental conditions allow at least a 30 min detection window for peptide chain elongation kinetics.

The dwell time of antibody binding (Δ*t*) is used to measure the total decoding time of 3xFLAG-Fluc mRNA. The histogram of *Δt* falls well within the 30+ minute detection window. The histogram fits to a log-normal distribution and yields an average of 3.9 ± 0.2 min (Figure 5B). Considering the 574-codon length of the 3xFLAG-Fluc coding region, the observed average elongation time corresponds to a peptide chain elongation rate of 2.5 ± 0.1 amino acids per second. This result is consistent with the previous estimate of 2.8–10 amino acids per second for *in vivo* budding yeast translation (50).

Several features of our experimental condition contribute to allow this long detection window for the decoding process: (i) the antibodies have a high labeling ratio of 2–7 dyes per antibody; (ii) Cy3 is photostable and (iii) this assay only requires a very low laser excitation (10 μW at the objective) and a slow data acquisition speed (2 s per frame).

### General applicability for studying initiation kinetics in other *in vitro* translation systems

**Figure 6.**
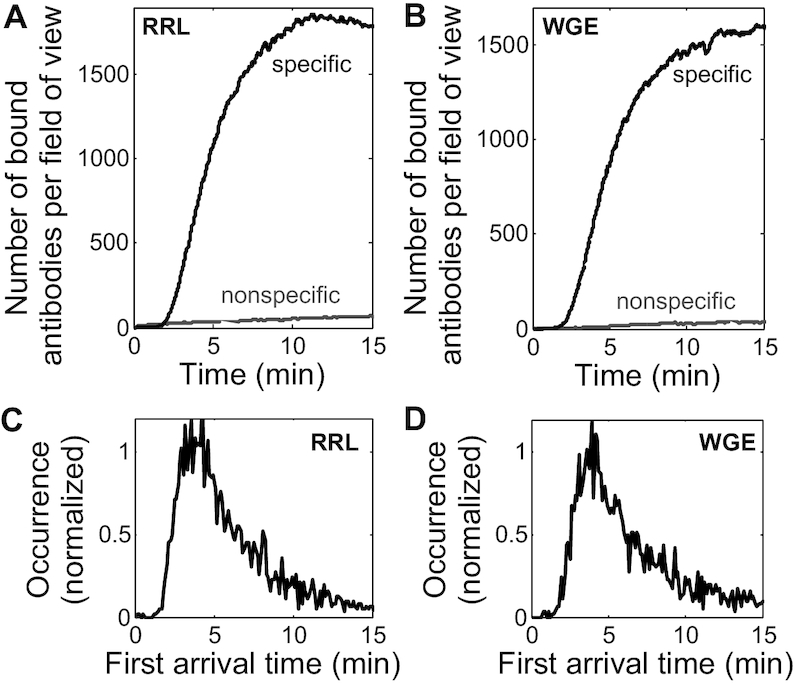
Cy3-antiFLAG binding in wheat germ extract (WGE) and rabbit reticulocyte lysate (RRL) translation reactions. (A, B) Representative time courses of antibody binding to Fluc (gray) and 3xFLAG-Fluc (black) mRNAs in RRL (**A**) and WGE (**B**) translation reactions. Data are representative of multiple experiments per condition: 2 (RRL specific binding), 3 (RRL nonspecific binding), >80 (WGE specific binding), 16 (WGE nonspecific binding). (**C**, **D**) Normalized first arrival time histograms for the experiments in (A) and (B), respectively. *n* = 3094 trajectories for RRL, and 3245 trajectories for WGE. Both histograms fit well to the shifted (3-parameter) log-normal distribution (Equation 1, Supplementary Figure S6, and Supplementary Table S1).

To test the applicability of our single-molecule method to other *in vitro* translation systems, rabbit reticulocyte lysate (RRL) and wheat germ extract (WGE) were utilized with our single-molecule assay. We found that the optimal experimental conditions for YE also worked well for RRL (Figure 6A). Interestingly, WGE exhibited a low level of nonspecific binding even under conditions that are more prone to nonspecific binding, such as with a higher laser power of 20 μW and a higher antibody concentration of 134 nM (Figure 6B). A typical *SNBR* of >25 was observed for RRL and WGE under these experimental conditions. The histograms of the first arrival time for both RRL and WGE (Figure 6C and D) showed an asymmetric distribution with a long tail, which is qualitatively similar to the first arrival time distribution for YE (Figure 4B) and fits well to the shifted (three-parameter) log-normal distribution (Supplementary Figure S6C and D, Supplementary Table S1). These results indicate that our assay is compatible with diverse translation extracts, including those derived from fungal, plant, and animal cells.

## DISCUSSION

Here we demonstrated a single-molecule strategy for characterizing *in vitro* eukaryotic cap-dependent translation initiation kinetics by measuring Cy3-antiFLAG binding to nascent N-terminal 3xFLAG peptides during active translation. To our knowledge, this is the first *in vitro* single-molecule assay that can track individual eukaryotic cap-dependent initiation events. Our assay differs from typical single-molecule TIRF experiments in several ways, such as the use of cell extracts rather than purified components, a very high concentration of diffusing fluorescent molecules, very low laser illumination, a large laser incident angle, and a double-PEGylated detection surface. Our standard experimental conditions worked well with YE, RRL and WGE, and WGE functioned well even under a condition that is more prone to nonspecific binding. We did not test this assay with yeast or mammalian translation systems that are reconstituted with purified components (6,7). But we expect compatibility with such systems because purified proteins are usually much easier to work with than cell lysates for single-molecule detection (51). Therefore, our single-molecule strategy should be generally applicable to existing *in vitro* eukaryotic translation systems.

Importantly, the single-molecule condition in this assay preserved the same translation kinetics as the bulk *in vitro* translation assays (Supplementary Figure S1). Our quantitative analysis focused on two basic parameters of antibody binding kinetics, the first arrival time (*t*_1_) and the dwell time (Δ*t*). These two parameters report on the initiation and peptide chain elongation kinetics, respectively. The dwell time analysis (Figure 5B) yielded an average peptide chain elongation rate of 2.5 ± 0.1 a.a./s for YE, consistent with previous *invivo* estimates of 2.8–10 a.a./s for budding yeast (50). The recent *in vivo* single-molecule translation assays, all carried out in human cell lines and neurons, reported an average peptide chain elongation rate of 3–10 a.a./s (14–17). The first arrival time analysis for all three types of extracts used (Figures 4 and 6) revealed that the first round of initiation occurs on the order of minutes. So far, quantitative measurements of initiation kinetics are very limited. The *in vivo* single-molecule translation assays (15–17) also provided minute-scale estimates for cap-dependent initiation. Bulk luminescence kinetics provides a convenient way for characterizing the overall translation kinetics, but lacks the resolution to dissect the initiation kinetics. Specifically, Berthelot *et al.* (11) showed that *in vitro* translation of Firefly luciferase encoding mRNAs in the budding yeast translation extract started to produce detectable luminescence in 8 to 20+ min for 5′ UTR lengths ranging from 43 to 1770 nt. Vassilenko *et al.* (12) showed that *in vitro* translation of Firefly luciferase encoding mRNAs in both wheat germ and Krebs-2 translation extracts started to produce detectable luminescence in 6+ minutes for 5′ UTR lengths ranging from 5 to 912 nt. These two studies provide the most quantitative and systematic bulk characterizations of cap-dependent translation kinetics so far. Consistent with Berthelot *et al.*, in our YE translation of the 3xFLAG-Fluc mRNA with 177 nt 5′ UTR, luminescence started to appear at round 9 minutes (Supplementary Figures S1 and S5). Although determining the initiation time from those two bulk luminescence kinetics measurements is not feasible, their agreement with our luminescence kinetics should support a similar time scale for initiation. Overall, we and the above-mentioned studies observe first round initiation typically occurring on the order of minutes for cap-dependent translation, regardless of mRNA 5′ UTR length. Collectively, these studies support the long-standing belief that initiation is the rate-limiting step in cap-dependent translation. It is worthwhile to point out that the single-molecule trajectories contain much more kinetic information than what we have discussed here. The strategy for a more comprehensive kinetic analysis depends on the central questions for each specific study.

Messenger RNAs with long coding regions can often be translated by multiple ribosomes simultaneously (‘polysome formation’). We indeed observed polysome formation in our experiments (Figure 1C bottom panel as an example trajectory). First arrival time analysis, by definition, does not depend on whether polysomes can form. However, polysome formation may complicate the dwell time analysis. On trajectories with polysome translation events, it may be challenging to correctly assign the time points of arrival and dissociation for individual antibodies, making it difficult to determine dwell times of single ribosome translation. Therefore, peptide chain elongation kinetics is often calculated solely from isolated single ribosome (‘monosome’) translation events. Under polysome-dominated translation conditions, the low amount of monosome events can impede robust dwell time analysis. A straight-forward strategy to deal with this problem is to use a mixture of labeled and unlabeled antibodies, therefore, only a fraction of ribosome translation events will be detected by labeled antibody binding. In this case, the actual translation activity is not perturbed but the single-molecule trajectories will be greatly enriched with monosome events.

Although we only used multiple Cy3-labeled antiFLAG in this paper, the idea underlying this assay should be compatible with other antibody/epitope pairs and fluorophores. The property of the antibody/epitope pairs and fluorophores can affect the performance of this assay in various aspects, and therefore should be carefully evaluated similar to our analyses in Figures 2, 3 and 5. Furthermore, given the excellent performance of the multiple Cy3-labeled antiFLAG in our experiments, we expect this antibody to be a good choice for a wide range of applications. The superior sensitivity and specificity of 3xFLAG/antiFLAG binding in antibody detection applications is widely recognized, therefore not examined here. We will focus our discussion on the optical properties of this antibody labeled with multiple Cy3 dyes and their impact on this assay. Fluorophore blinking is a common problem in single-molecule fluorescence experiments and if severe, can greatly complicate or impede accurate kinetic analysis. The single-molecule trajectories in our experiments did not show evidence of blinking, likely benefiting from the multiple labeling, the seconds-scale data acquisition speed, and the very gentle laser excitation condition. Photobleaching is another common complication in single-molecule experiments. Since photobleaching increases with stronger laser excitation, the very gentle laser excitation used in this assay is beneficial for minimizing photobleaching. Furthermore, due to the fast exponential decay of laser excitation energy in TIRF imaging, only fluorophores that stay close to the detection surface are susceptible to photobleaching. Accurate first arrival time determination is relatively insensitive to photobleaching because it only requires reliable measurement of the arrival of diffusing antibodies to the surface-tethered mRNA/ribosome/peptide complex. However, after antibody binding, the observed dwell time can be artificially shortened if photobleaching is severe. Under our experimental conditions, Cy3-labeled antiFLAG was calibrated to remain photostable for over 30 minutes (Figure 5A). Given the peptide chain elongation rate observed with our single-molecule system (Figure 5B), and with other bulk studies, even a several kb long coding region will typically take only a few minutes to translate. Therefore, the multiple Cy3-labeled antiFLAG should enable studies of cap-dependent translation with a wide range of open reading frame sequences and translation systems beyond what we have used here.

A common assumption underlying rate constant determination from bulk kinetics measurements is that the reaction progresses with a high degree of synchronization among the entities of interest. Specifically in the translation rate determination from bulk luminescence measurements (11,12), most mRNAs should go through the initiation and peptide elongation stages with similar pace. However, the validity of this assumption has not been tested so far. In our assay, the starting point of the translation reaction was synchronized by the delivery of translation reagent (Figure 1C), yet the histograms of the first arrival time for all three extracts that we tested exhibited the same qualitative feature of asymmetrical distribution over a large span of time. Specifically for YE translation of 3xFLAG-Fluc mRNA, the first antibody binding per mRNA occurred as early as 2 min and as late as 30+ min after flowing in the translation reagents (Figure 4B). In contrast, the average peptide elongation time of the entire coding region is only ∼4 min (Figure 5B). Therefore, the mRNAs that start translation early (‘early starter mRNAs’) can have multiple rounds of protein synthesis before the ‘late starter mRNAs’ begin their first round of peptide synthesis. Such differences in translation kinetics were indeed observed in the example trajectories of early starter mRNAs (Figure 1C) and late starter mRNAs (Supplementary Figure S7). To assess the extent of this asynchrony between mRNA molecules, we divided the cumulative histogram of the first arrival time into 14 intervals with an even spacing equal to the average peptide elongation time (Supplementary Figure S8). On average, the mRNA molecules in an interval will lead the molecules in the subsequent interval by one cycle of peptide synthesis. As can be seen in Supplementary Figure S8, 30%, 22%, and 13% of the mRNA molecules fall in the first, second and third interval, respectively. The remaining 35% of the mRNAs start peptide synthesis in either the fourth or a later interval. This observation is in line with the common belief that initiation is the rate limiting step in cap-dependent translation and, importantly, highlights an unrecognized high degree of intrinsic translational asynchrony between mRNA molecules. Our results indicate that translation kinetic results should be interpreted with caution to avoid over-simplified assumptions about translation kinetics. As cap-dependent initiation is a multiple step process involving dozens of initiation factors, understanding the molecular nature of this translational asynchrony will require a systematic investigation that combines our single-molecule assay with yeast mutagenesis.

Slight variation in conditions between repeated experiments is inevitable, even with meticulous attention to details. In our hands, we found that first arrival time kinetics between multiple repeats (Figure 4A) are significantly more robust than the bulk luminescence kinetics (Supplementary Figure S5). Our observation of the striking asynchrony between mRNA molecules in translation activity suggests that mathematically the total protein yield over time depends on the kinetics of initiation, peptide elongation, and other translation steps in a convoluted fashion. Therefore, the bulk luminescence kinetics may be more sensitive to stochastic variations in experimental conditions than first arrival time. When comparing YE translation of two mRNAs that differ only by the insertion of a small hairpin structure (Δ*G* = −4.8 kcal/mol) positioned in the middle of the 5′ UTR (Figure 1A), our assay robustly detected slower initiation kinetics with the mRNA containing the hairpin structure (Figure 4). However, we found it very challenging to resolve this difference by bulk luminescence kinetics (Supplementary Figure S5). Consistent with this, RNA structures with such low thermostability were considered insufficient to affect initiation in previous bulk measurements (48). Low assay robustness clearly contributes to the difficulty of detecting small changes in initiation kinetics with bulk luminescence kinetics. In addition, the complicated dependence on translation kinetics might also render the bulk luminescence kinetics to be less sensitive to small differences in initiation kinetics. The single-molecule resolution and more direct measurement of initiation events are two key factors that allow our assay to be more robust and sensitive to initiation kinetics than bulk luminescence-based approaches.

Despite the high degree of asynchrony between the translation activity on individual mRNA molecules, the single-molecule resolution in our assay offers a straight-forward way to determine initiation kinetics from antibody binding kinetics. For each antibody binding event, biochemically we know that *t*_1_ = *t*_1_tag_ + *t*_1_I_. As initiation and peptide elongation are two separate molecular processes involving different translation factors, *t*_1_tag_ and *t*_1_I_ are two independent random variables. Therefore, the average of all three variables have the simple relation that <*t*_1_> = <*t*_1_tag_> + <*t*_1_I_>. In translation initiation studies, the peptide synthesis conditions (such as coding region sequence and component concentrations) are usually purposely preserved. Furthermore, many studies focus on the difference in <*t*_1_I_> between different translation conditions. Therefore, it is likely suitable for many experiments to directly work with <*t*_1_>. When quantification of the actual initiation time is needed, <*t*_1_tag_> can be calculated and subtracted from <*t*_1_>. Specifically, <*t*_1_tag_> can be calculated as the product of the average peptide chain elongation rate, determined from dwell time analysis, and the total length of the 3xFLAG tag and the segment of nascent peptide protected by the ribosome exit tunnel. The protected nascent peptide length ranges between 30–40 amino acids because different nascent peptide sequences can form different structures inside the ribosome exit tunnel (45). The 10 amino acids range corresponds to less than 5s difference for the average peptide chain elongation rate observed in our YE translation and other studies. In comparison to the minutes-scale initiation rate, it may be sufficient to use an estimated length of ∼35 a.a. for the protected nascent peptide. In case that higher accuracy is needed, reporter mRNA may be designed to contain the 3xFLAG sequence followed by a peptide sequence with a characterized protected length. This assumption-free calculation of the initiation time is a significant advantage of our assay over the bulk kinetic approaches (11,12) or the recent *in vivo* single-molecule translation assays (14–17).

The ability of our single-molecule assay to resolve individual ribosome-mediated translation events creates new opportunities to better understand the molecular mechanisms of cap-dependent initiation from a kinetics perspective. For example, kinetic modeling of the timing that corresponds to the multiple initiation events per mRNA should provide insights into the effects of scanning ribosomal particles on mRNA 5′ end sequestration from subsequent ribosome binding. When combined with mutations in initiation factors, such kinetic analyses can be used to study the molecular mechanisms of specific initiation factors and their effects on the initiation process. Furthermore, although here we focused on canonical cap-dependent translation initiation, this assay should be easily adaptable to many non-canonical initiation mechanisms, such as m^6^A mediated (52) or 3′ cap-independent translational enhancers (3′ CITEs) (53) stimulated initiation. In many cases, the effort to adapt an existing *in vitro* system of a non-canonical initiation mechanism to our single-molecule assay should simply involve cloning of the 3xFLAG sequence to the N-terminus of a reporter and biotinylation of the reporter mRNA 3′ end.

## Supplementary Material

gkz1066_Supplemental_File
