# Supplementary material for: An in vitro single-molecule assay for eukaryotic cap-dependent translation initiation kinetics
Source: Nucleic Acids Res. 2019 Nov 13;48(1):e6. doi: 10.1093/nar/gkz1066 (PMC7145701; doi:10.1093/nar/gkz1066)
Supplement: gkz1066_Supplemental_File [file gkz1066_supplemental_file.pdf]

SUPPLEMENTARY TEXT

The binding ratio of anti-FLAG to 3xFLAG peptide

The 3xFLAG sequence was chosen over the 1xFLAG sequence in this assay because anti-FLAG detection of 3xFLAG peptide has 10-fold higher sensitivity than 1xFLAG peptide detection in bulk applications. The three repeats of the FLAG sequence raise the possibility of multiple antibodies binding to the same peptide. To address this, we characterized the trajectories of YE translation of 3xFLAG-Fluc mRNA into three categories: I) single antibody binding at a time, such as the top two example trajectories in Figure 1C; II) multiple antibodies bind sequentially and dissociate sequentially, such as the bottom example trajectory in Figure 1C; III) multiple antibodies bind sequentially and dissociate simultaneously. Except for trajectories that are too noisy or have too many antibody binding events to unambiguously resolve individual binding steps, all trajectories with clearly resolvable antibody binding steps fall under these three categories. The type I trajectories clearly correspond to the case of single antibody binding per peptide. As discussed in the main text (Figure 5A), fluorophore photobleaching or instability of Cy3-antiFLAG/3xFLAG interactions cause very little premature fluorescence loss over the time scale of peptide chain elongation; rather, loss of fluorescence predominantly results from the dissociation of the Cy3-antiFLAG/nascent peptide complex from the ribosome and mRNA upon translation termination. Therefore, type II trajectories correspond to the scenario of multiple ribosomes translating the same mRNA with a single antibody binding per peptide. Among the three categories, only type III trajectories may be interpreted as translation of an mRNA by a single ribosome with multiple antibodies bound to the nascent peptide. Overall, we found that 73±3% (s.d.), 15.8±0.3% (s.d.), and 11±3% (s.d.) of trajectories with clearly resolvable antibody binding steps fall under categories I, II, and III, respectively, indicating that approximately 90% of nascent peptides are bound by only one antibody. Therefore, the 3xFLAG tag predominantly recruits one anti-FLAG in our assay.

SUPPLEMENTARY FIGURE 1

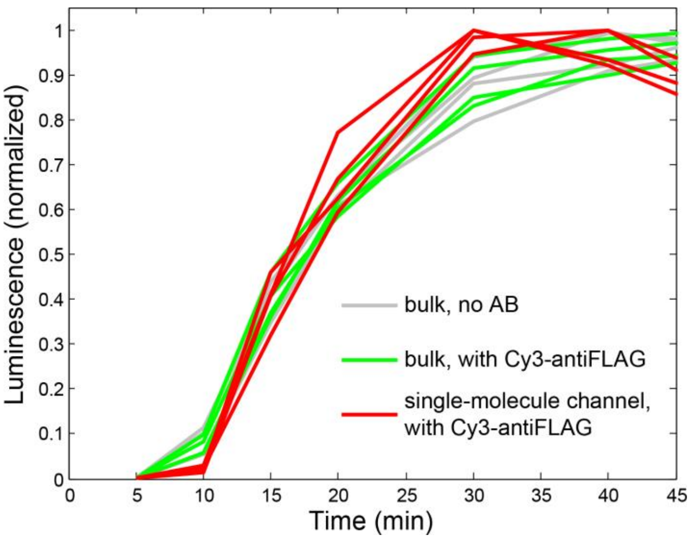

Figure S1. Luminescence kinetics for YE translation of 3xFLAG-Fluc mRNA in bulk (gray and green) and single-molecule (red) conditions. The bulk conditions were carried out following our standard YE *in vitro* translation protocol in a microcentrifuge tube, without (gray) or with (green) Cy3-antiFLAG supplementation. The single-molecule condition was carried out as illustrated in Figure 1 with mRNAs tethered to the detection surface via the 3' end. At selected time points, an aliquot of the translation mixture was pipetted out from either condition for luminescence reading. The y axis is normalized to the plateau value of each curve. Each condition was repeated four times.

SUPPLEMENTARY FIGURE 2

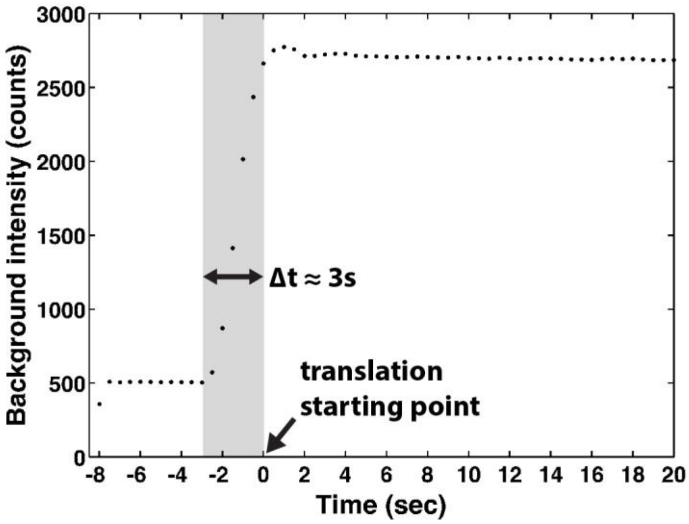

Figure S2. A representative trace for the background fluorescence intensity change as the translation mixture containing Cy3-antiFLAG is introduced into the flow channel. The gray area highlights the buffer exchange process, which takes approximately 3 seconds to complete for this data set, and typically 3-4 seconds for all data sets. The arrow indicates the completion of buffer exchange, which also sets the starting point of the translation reaction.

SUPPLEMENTARY FIGURE 3

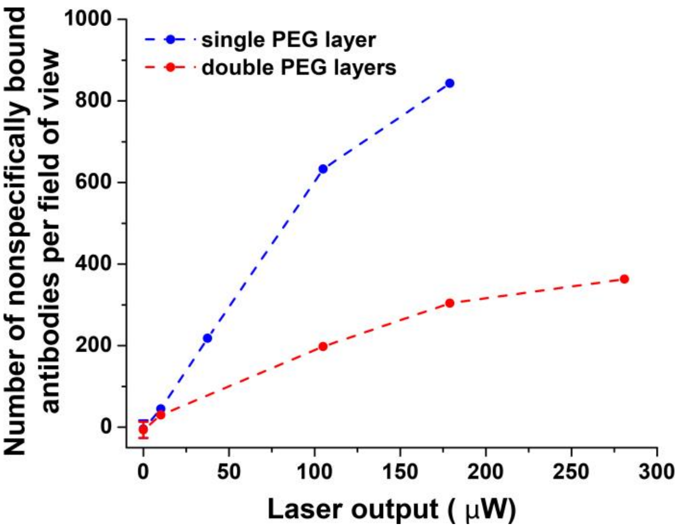

Figure S3. The effect of laser illumination and surface passivation on nonspecific Cy3-antiFLAG binding. YE translation of surface-immobilized Fluc mRNA in the presence of 67nM Cy3-antiFLAG was used to measure antibody nonspecific binding. The number of nonspecifically bound Cy3-antiFLAG per field of view after 10 minutes of continuous laser excitation was plotted against the laser power measured at the objective for both single- (blue) and double- (red) PEGylated surfaces.

SUPPLEMENTARY FIGURE 4

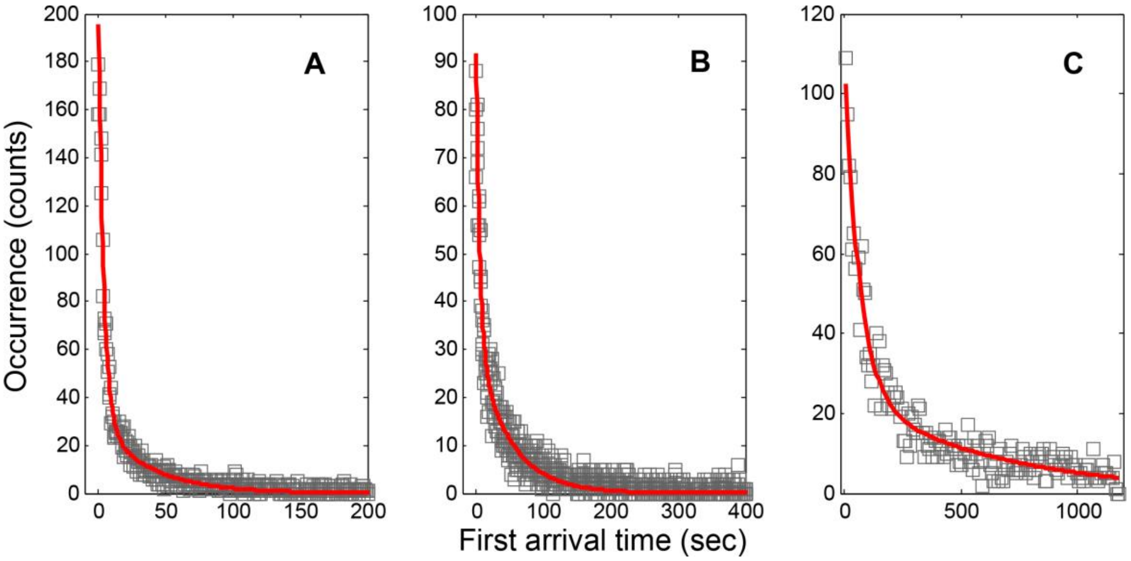

Figure S4. Kinetics of Cy3-antiFLAG recognition of pre-existing nascent 3xFLAG-Fluc peptides with 67 nM (A), 22 nM (B) and 7 nM (C) antibody concentrations. The experimental procedure was the same as for Figure 3 (black curve), except for the antibody concentrations. (A) is the same data as Figure 3 (black curve), except that x axis is in linear scale. n = 4952 trajectories (B) and 2470 trajectories (C). Similar to Figure 3 (black curve), the 22-nM histogram (B) was fit to a double exponential distribution and the time constant of antibody binding to pre-existing nascent 3xFLAG peptides was determined from the faster exponential component as 5.6 ± 0.5 (s.e.) seconds. For the 7-nM condition (C), the time constants of the two exponential components are too close to be robustly determined by double exponential fitting. Therefore, the histogram was fit to a single-exponential distribution, which yielded a time constant of 64 ± 7 (s.e.) seconds. R^2 = 0.9822, 0.9496, and 0.9508 for (A), (B), and (C), respectively.

SUPPLEMENTARY FIGURE 5

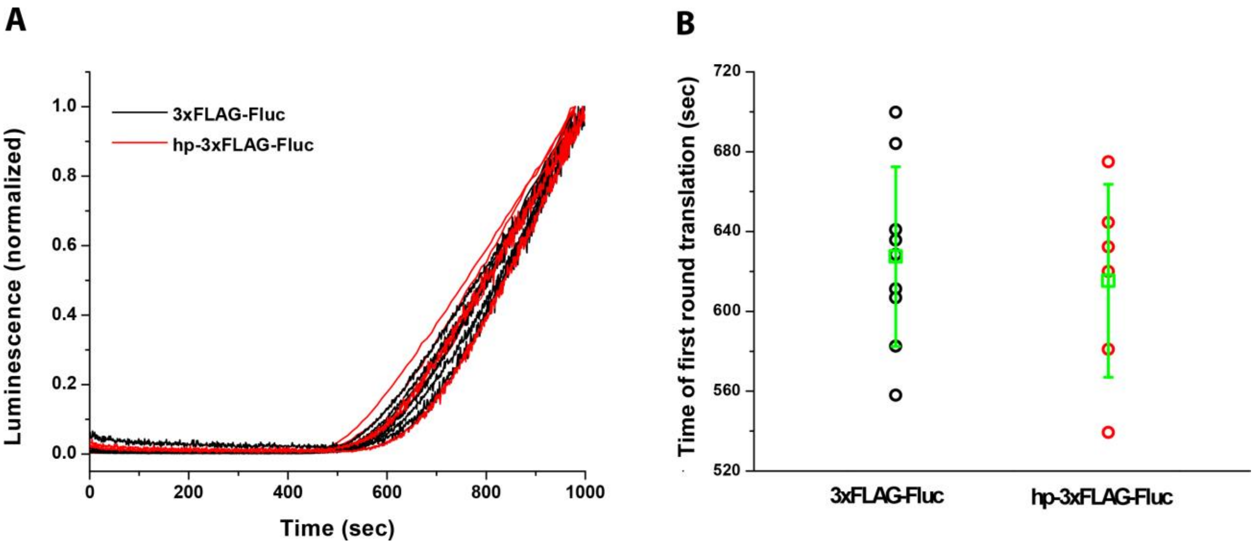

Figure S5. Kinetics of YE translation of 3xFLAG-Fluc (black; 9 data sets) and hp-3xFLAG-Fluc (red; 6 data sets) mRNAs by bulk luciferase activity assay. **A**: Luminescence kinetics. **B**: Scatter plots of first round translation times determined by Gaussian fitting to the second derivative of the luminescence kinetics curves in (**A**), as described by Vassilenko *et al*. The green boxes and error bars in (**B**) represent the mean value and standard deviation of the calculated first round translation time, respectively. Due to the experimental variations, the two mRNAs do not show a statistically significant difference in translation kinetics.

SUPPLEMENTARY FIGURE 6

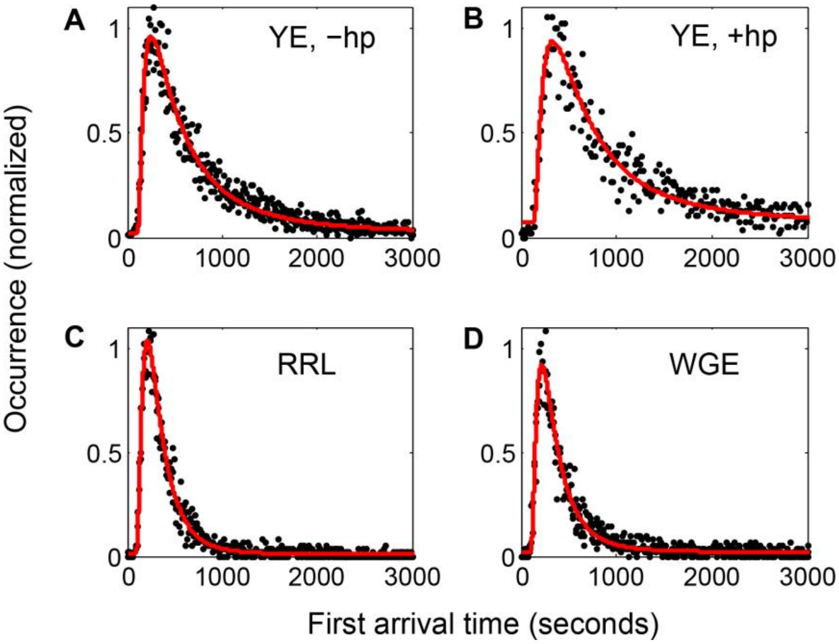

Figure S6. Shifted (3-parameter) log-normal fitting (red) of first arrival time histograms (black) for all translation conditions. A, B: YE translation of 3xFLAG-Fluc (**A**) and hp-3xFLAG-Fluc (**B**) mRNAs. **C, D**: RRL (**C**) and WGE (**D**) translation of 3xFLAG-Fluc mRNA. The data are the same as in Figures 4B, 6C, and 6D. The fit results are summarized in Table S1.

SUPPLEMENTARY FIGURE 7

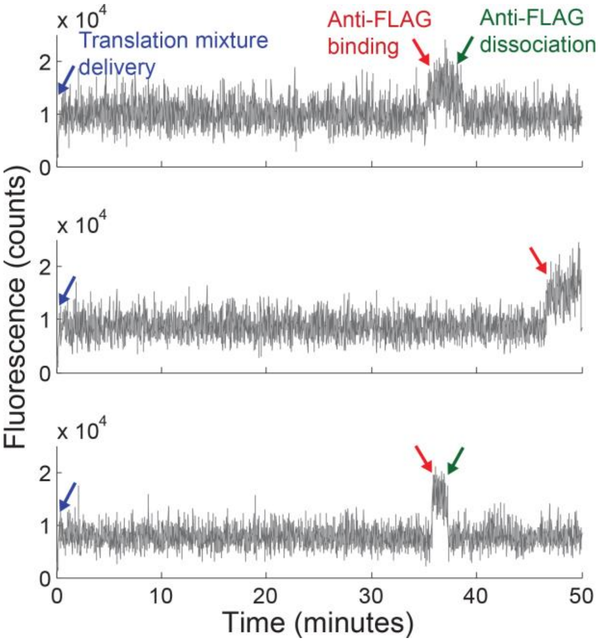

Figure S7. Example trajectories of mRNAs that start the first-round of translation very late. The blue, red, and green arrows indicate the moment of translation mixture delivery, anti-FLAG binding, and anti-FLAG dissociation, respectively. The trajectories are from the same experiment as the example trajectories in Figure 1C.

SUPPLEMENTARY FIGURE 8

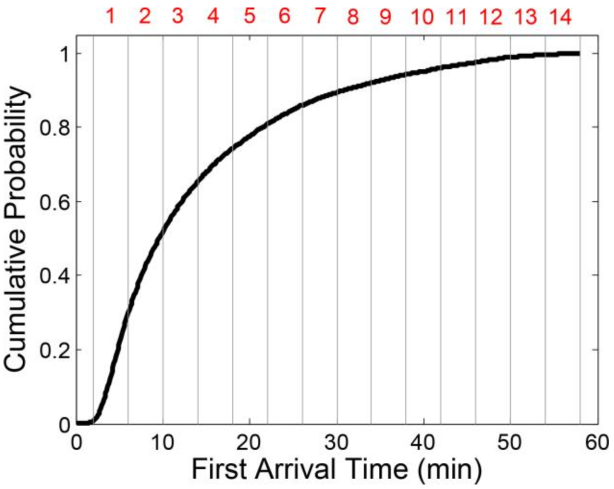

Figure S8. Cumulative probability of the first arrival time for YE translation of 3xFLAG-Fluc mRNA. The plot was built with the same data used for Figure 4B (black curve). The vertical grey lines indicate the breaking down of the total time range in intervals of 4 minutes (the average peptide synthesis time for the whole coding region). The first line sits at 2 minutes, when the earliest antibody binding events are observed.

SUPPLEMENTARY TABLE 1

Summary of shifted log-normal distribution fitting of the first arrival time histograms for all translation conditions

	**mRNA**	**Translation Extract**	**x_0 ± s.e.** (s)	**μ ± s.e.** (ln(s))	**σ ± s.e.** ($(\ln(s))^{1/2}$)	**Adjusted R-Square**	**Number of data**
1	3xFLAG -Fluc	YE	108 ± 3	6.07 ± 0.03	1.04 ± 0.03	0.95	3 data sets; 10650 trajectories in total
2	hp-3xFLAG-Fluc	YE	131 ± 9	6.29 ± 0.05	0.99 ± 0.05	0.91	2 data sets; 4079 trajectories in total
3	3xFLAG -Fluc	RRL	83 ± 3	5.4 ± 0.01	0.73 ± 0.02	0.97	1 data set; 3094 trajectories
4	3xFLAG -Fluc	WGE	97 ± 4	5.43 ± 0.02	0.79 ± 0.02	0.95	1 data set; 3245 trajectories

1. The specific functional form used for fitting is shown in Equation 1.

2. Consistent with the low ratio of nonspecific to specific antibody binding under our experimental condition, the ratio of y_0 to A in Equation 1 is smaller than 10^{-3} for all above data sets.
